# N-Type Thermoelectric Performance of Functionalized Carbon Nanotube-Filled Polymer Composites

**DOI:** 10.1371/journal.pone.0047822

**Published:** 2012-11-02

**Authors:** Dallas D. Freeman, Kyungwho Choi, Choongho Yu

**Affiliations:** Department of Mechanical Engineering, Texas A&M University, College Station, Texas, United States of America; Queen's University at Kingston, Canada

## Abstract

Carbon nanotubes (CNTs) were functionalized with polyethyleneimine (PEI) and made into composites with polyvinyl acetate (PVAc). CNTs were dispersed with different amounts of sodium dodecylbenzenesulfonate (SDBS) prior to the PEI functionalization. The resulting samples exhibit air-stable n-type characteristics with electrical conductivities as great as 1500 S/m and thermopowers as large as −100 µV/K. Electrical conductivity and thermopower were strongly affected by CNT dispersion, improving the properties with better dispersion with high concentrations of SDBS. This improvement is believed to be due to the increase in the number of tubes that are evenly coated with PEI in a better-dispersed sample. Increasing the amount of PEI relative to the other constituents positively affects thermopower but not conductivity. Air exposure reduces both thermopower and conductivity presumably due to oxygen doping (which makes CNTs p-type), but stable values were reached within seven days following sample fabrication.

## Introduction

Functional thermoelectric devices can produce electricity anywhere there is a temperature gradient. Conversely, they can be employed as refrigeration devices if current is supplied, cooling without the use of pumps or fluids. At this point, the limited efficiency of thermoelectric modules restricts their applications in both electricity generation and refrigeration to situations where longevity, space requirements, and quiet operation are of principle importance [1].

Starting in the 1990s, research into thermoelectric materials has been re-energized by new material fabrication techniques. By controlling the structure at the nano- and micro-scales, improvements have been made to the efficiency of the Bi-Te alloys, which were the state of the art for decades [2]. It is hoped that further research into nanomaterials will broaden the applicability of thermoelectric energy conversion by enhancing their performance.

The thermoelectric figure of merit, used to describe the thermoelectric effectiveness of any material, is given by 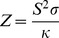
(1)where *S*, *σ*, and *κ* are thermopower, electrical conductivity, and thermal conductivity, respectively [2]. Polymers typically have low thermal conductivity, which is a good starting point for a high figure of merit. However, typical polymers do not have an electrical conductivity, high enough for efficient thermoelectrics. In order to improve the electrical properties, carbon nanotubes have been used together with polymers to synthesize composites [3], [4], [5], [6], [7].

Carbon nanotubes (CNTs) have been extensively studied as potential solutions in a wide range of applications due to their unique mechanical, electrical, and geometric properties [8]. Many of the tubes, which are composed of one or more rolled sheets of the carbon honeycomb structure known as graphene, are either metallic or semiconducting, depending on the lattice vector by which they are rolled. Intrinsically n-type semiconducting nanotubes are highly susceptible to oxygen doping and become p-type in atmosphere [9], [10].

Several methods have been demonstrated for the production of air stable n-type nanotubes, including passivation of a protective film around the tubes to prevent oxygen doping, application of viologens for a direct redox reactions, and the use of metal electrodes with low work functions [11], [12], [13]. A simpler production method has also been demonstrated wherein the physical adsorption of branched polyethyleneimine (PEI) onto carbon nanotubes results in a conversion of the conducting properties from p-type back to n-type [6], [14]. Nanotubes functionalized with PEI have been used to produce p-n junctions, photovoltaic cells, and field-effect transistors [14], [15], [16], [17]. In our previous work, the thermopower of thin films composed of PEI-doped tubes was measured to be as large as -60 µV/K [6]. Such films, composed almost purely of carbon nanotubes, are not prime candidates for thermoelectric generation because their thermal energy transport may be too high due to their intrinsically high thermal conductivity [10].

CNTs were combined with polymers to synthesize composites, and their electrical and thermal properties have been evaluated in our prior work [3], [4], [5], [18]. Despite typical correlation between thermal and electrical conductivities, these composites exhibit electrical conductivities nearly as high as films composed exclusively of tubes, but still possess thermal conductivities closer to those their polymer matrices. This phenomenon results from the relative ease with which charge carriers travel across the nanotube networks by hopping. The thermal carriers, or phonons, have relative difficulty with transport because they are scattered at the CNT surfaces and at the junctions between the tubes [3]. The result is a material with high electrical conductivity and low thermal conductivity. For example, composites with p-type doped CNTs exhibited electrical conductivities as high as ∼10^5^ S/m [3]. While several studies have been conducted on polymer composites containing CNTs, air-stable n-type polymer composites with CNTs have never been reported. The overall aim of this study was to produce and test such composites and to determine the conditions which result in the best thermoelectric performance.

## Results and Discussion

**Figure 1:**
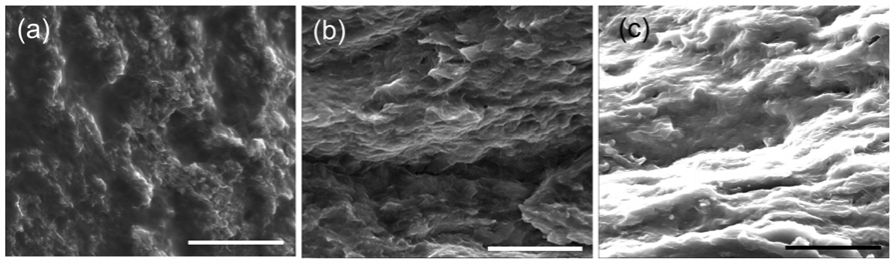
SEM images of cold fractured surfaces for samples with 20, 40, and 60-wt% SDBS (a, b, and c, respectively). These samples contain 20 wt% CNTs (99% purity). Fractures become sharp and disorganized as less surfactant is included, eventually forming heterogeneous structures as seen in (a). Scale bars indicate 10 µm.

Series 1 was composed of samples containing 20-wt% CNT, 10-wt% PEI, and a varied amount of sodium dodecylbenzenesulfonate (SDBS). As shown in the scanning electron microscope (SEM) images for three samples of different conductivities (Figure 1), the samples with higher SDBS weight percent exhibit smoother cleavages and fewer CNT pullouts (PEI wraps around CNTs, it is rather hard to see CNTs clearly in the micrographs). Both of these are characteristics of good dispersion, indicating that SDBS weight percent at a ratio of 3 to 1 with CNT is more effective at deconstructing the bundles than the smaller ratios. As the amount of SDBS goes down, the CNTs agglomerate more, forming networks around internal voids like those shown in Figure 1a. Such voids reduce overall electrical conductivity for the composite.

**Figure 2:**
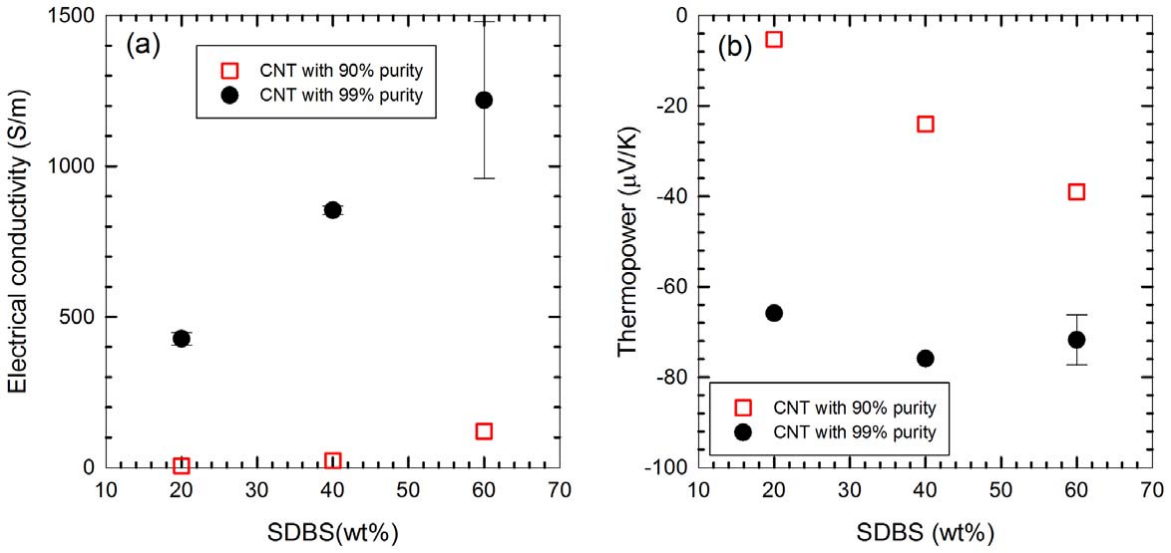
Electrical conductivity (a) and thermopower (b) for Series 1 samples containing 20-wt% CNT, 10-wt% PEI, and a varied amount of SDBS. Correlation is positive for thermopower magnitude and electrical conductivity.

Figure 2 depicts correlation between SDBS and both thermopower and electrical conductivity. There are large differences between tubes rated as 99% and 90% purities, probably due to the lower conductivity of amorphous carbon and the catalyst impurities [7]. The heavy catalyst particles may also have a negative effect on dispersion, forcing tubes to which they are attached to fall into a precipitate. It also clearly shows strong dependence of the properties as a function of SDBS concentrations. In most materials, thermopower and conductivity have an inverse relationship, explained qualitatively by the fact that an increased amount of charge carriers will drive down the voltage potential being induced by the temperature gradient. However, our results show an increase in electrical conductivity resulted in a larger thermopower (absolute value). We believe that this can be attributed to CNT dispersion, which makes the sites for PEI doping upon better dispersion with a high concentration of SDBS.

The electrical conductivity of the PEI-doped samples (∼10^3^ S/m) in this study is lower than that of the samples that we previously reported [3], [4], [5]. This can be attributed to the poor electrical conductivity (an electrical insulator [19]) and the branched nature of PEI. When CNTs are embedded in the composite, PEI wraps around CNTs, hindering direct contacts between nanotubes. The tube-tube junctions play an important role in altering the electrical conductivity of such composites [5]. When electrically-conducting poly(3,4-ethylenedioxythiophene) poly(styrenesulfonate) (PEDOT:PSS) is used for dispersing CNTs, they are electrically well connected due to the presence of electrically conducting PEDOT:PSS at the junctions. Another reason is that the current study includes only 20 wt% (except one 40-wt% sample) CNTs, as opposed to ∼60 wt% CNT concentration to achieve 10^5^ S/m conductivity. Additionally, we used CNTs synthesized by a chemical vapor deposition method, which have relatively low electrical conductivity compared to other tubes synthesized by an arc-discharge or high pressure carbon monoxide (HipCo) method [4], [7].

Dispersion remains a primary consideration in the production of CNT composites to obtain desired electrical, thermal, or/and structural properties [3], [4], [5], [7], [20]. In this work, the surfactant SDBS was chosen to facilitate dispersion based on its superior performance and for its tendency to form smooth coating layers around the CNTs [21], [22]. The principal challenge specific to this research resulted from the use of PEI. This polymer contains one of the highest densities of amine groups of all polymers, and donates electrons to the nanotubes [6], [23]. The amine groups contain electron lone pairs, which are responsible for the electron donation. This PEI attachment on CNTs makes CNTs electron-rich, converting p-type CNTs into n-type. When CNTs were well dispersed, the number of nanotubes in each bundle decreases, resulting in an increase in the amount of tubes coming into physical contact with the PEI molecules, allowing for more effective doping. In other words, fewer tubes from the center of the bundles are allowed to remain p-type and reduce the magnitude of the composite thermopower value, as discussed in the later sections. Conversely, when CNTs were “not” well dispersed, a reduction in electron pathways through the material happens. Fewer pathways mean that the composite as a whole will have less conductivity than a material where the tubes were more completely dispersed.

**Figure 3:**
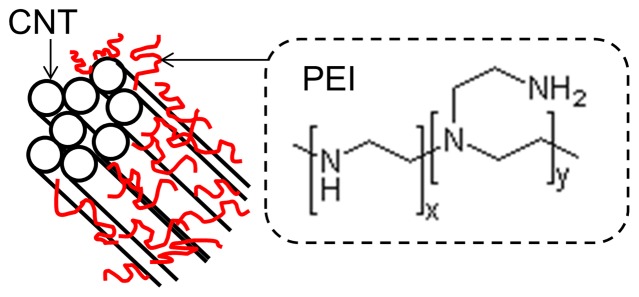
Schematic representation of PEI-functionalized CNT bundles in the composite and the structure of branched PEI. The amine groups are responsible for the electron donation which converts CNTs into n-type semiconductors. When the bundles are smaller, more tubes come in direct contact with PEI, which increases the n-type thermopower values. On the other hand, composites with large bundles have fewer junctions necessary for electron transport across the composite.

The PEI used in this study had an average molecular weight of ∼600, indicating that it was composed of molecules that were made of four to five units like the one shown in Fig. 3. Although covalent bonding has been demonstrated for the case of F-functionalized (fluorinated) CNTs, pristine CNTs like those used in this study interact with PEI dominantly via physisorption [7], [22] rather than chemisorption. In this process, the PEI molecules wrap around the CNTs and form bonds with other PEI molecules or with the opposite ends of the same molecule. It has been shown with atomic force microscope imaging that this coating of PEI molecules causes the tube to double in diameter relative to uncoated tubes [24]. In our experiments, when tubes were soaked in PEI solutions, and then filtered, rinsed, and allowed to dry, the product was heavier than the tubes prior to the process. In addition to adding mass, PEI also acts as a coagulant for CNTs in water which can counteract the effects of the surfactant. Visible tube agglomerations were observed when the ratio of PEI wt% to CNT wt% is higher than 0.5 during the PEI doping process. Such aggregation often makes the sample mechanically weak and/or hinders composite formation.

**Figure 4:**
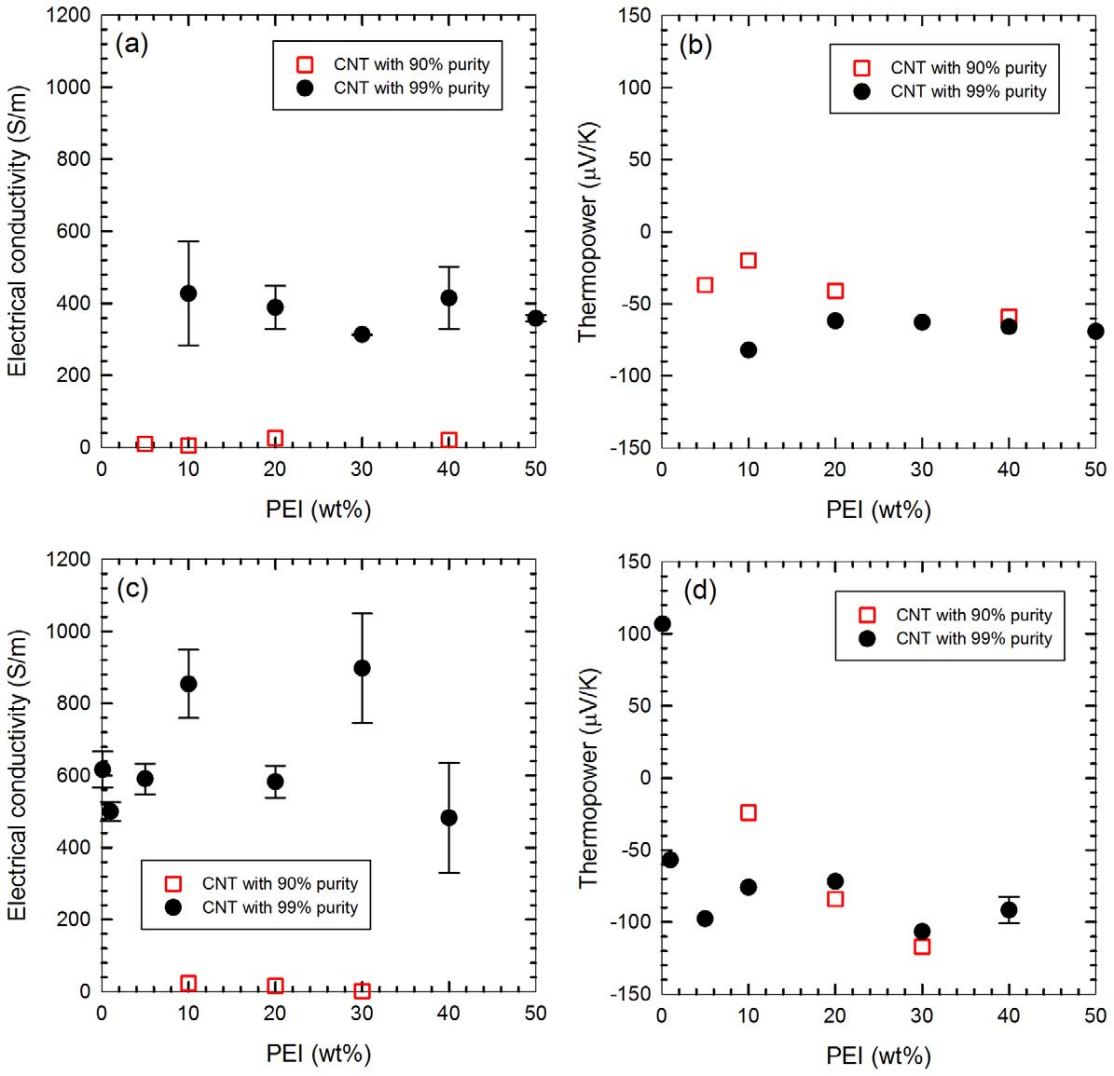
Effect of PEI wt% on the electrical properties for the samples containing 20-wt% (a and b: Series 2:) and 40-wt% (c and d: Series 3) SDBS. Thermopower sign changed with PEI whose concentrations between 0.1 and 1 wt%, as shown in (d), wherein it is seen that values for thermopower do not change significantly beyond 5-wt% PEI.

Series 2 and Series 3 varied the amount of PEI, being composed of 20-wt% CNT with 20-wt% (for Series 2) or 40-wt% (for Series 3) SDBS. When the higher SDBS concentration was used, electrical conductivity was more or less improved, presumably due to the better CNT dispersion. The data are somewhat scattered but the overall values for electrical conductivity are higher for the 40-wt% SDBS samples, compared to the 20-wt% samples. The change in thermopower values is rather small due to the small absolute values as well as the weighting factor (electrical conductivity) for n- and p-type thermopower of CNTs in the composite materials, as discussed more in detail below (see equation 2). The composites contain less SDBS compared to the best properties in Series 1 (60-wt% SDBS). As we had more CNTs compared to SDBS, it is much harder to disperse CNTs, which is a part of the reasons for the scattered properties. It should be noted that, unlike p-type properties, n-type strongly depends on PEI coverages. On the other hand, the samples exhibited little change with increasing PEI beyond 10 wt%, possibly indicating saturation (Figure 4). Beyond this point, it is likely that no rooms are available for additional PEI to attach.

The first three levels of Series 3 (0.1, 1, and 5-wt% PEI, in Figure 4(d)) demonstrate a correlation between PEI weight percent and thermopower. The sample containing only 0.1 wt% has a p-type thermopower comparable to control samples made without incorporating PEI. The 1-wt% sample exhibits n-type properties of a lower magnitude. Above 5 wt%, the samples demonstrate only a slight correlation with thermopower and additional PEI weight percent. Doping fractions by nitrogen per each carbon in CNTs were estimated. Assuming the structure of the branched PEI is shown in Figure 3 with x and y are 1 (i.e., 1 unit contains 3 nitrogen). Then, molecular weight of 5 units is ∼645, which is close to the specification from the manufacturer. The n-type conversion occurred when the ratio of CNT to PEI wt% is 1∶0.05 (i.e., 1 wt% PEI sample). In this case, the number of nitrogen per carbon can be calculated to be ∼3×10^−3^. In fact, this value is within the range, 1–6×10^−3^ [25] suggested by an experiment with a field-effect transistor configuration made of PEI-functionalized CNT. This may explain that relatively constant thermopower when PEI is more than 10 wt%.

**Figure 5:**
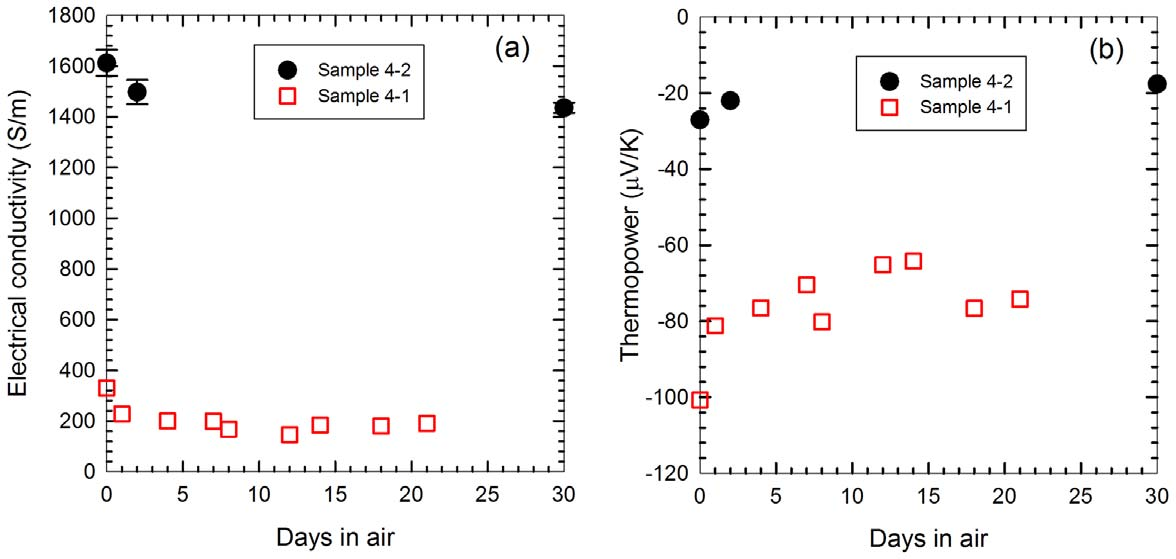
Change in electrical conductivity (a) and thermopower (b) as a function of time. The elapsed time was measured after the vacuum annealing (last process for fabricating samples) for Series 4 samples.

The effects of air on the samples (Series 4) over time are depicted in Figure 5. Both conductivity and thermopower decayed somewhat and then became more or less constant in the presence of air, owing to the increased levels of oxygen doping on the material. The simultaneous reduction in conductivity and thermopower experienced during atmospheric exposure over time can be understood in terms of a mixed carrier model, where a conductor uses both electrons and holes as charge carriers. In particular, each nanotube bundle in the composites is unlikely to have been permeated with PEI throughout the bundle. This will leave n-type tubes on the outside where the PEI is attached, while the tubes inside the bundle will still be p-type due to oxygen doping. In addition, as the sample is left exposed to the atmosphere, more oxygen molecules will infiltrate the polymer and remove more electrons from the tubes. Therefore, the entire composite is a mixture of p- and n-type charge carriers, which will recombine to have reduced carrier concentrations, resulting in a lower electrical conductivity. Assuming that both carriers are present after recombination, thermopower of a composite with mixed carriers may be described as [6]:
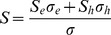
(2)where *S_e_* is the thermopower due to electrons (having a negative value) and *S_h_* is the value for holes; *σ_e_* and *σ_h_* are the conductivities due to electrons and holes, respectively; *σ* is electrical conductivity due to both electrons and holes. As can be seen, an increase in hole conduction will decrease the overall thermopower. CNTs also exhibit different electrical properties depending on their chirality, or the arrangement of the graphene hexagons relative to the tube axis [8]. These electronic differences may make individual tubes more or less responsive to doping, depending on metallic or semiconducting properties.

It should be noted that in the case for pristine n-type nanotubes in vacuum, air exposure results in an increase of conductivity, as oxygen donates sufficient holes to overcome the loss in electrons [9]. In our samples shown in Figure 5, after a few days of air exposure, thermopower was saturated. It appears the PEI coating sustains n-type doping for the time periods. This suggests PEI was coated on most of CNT surfaces, deterring oxygen doping.

In the future, studies could be conducted to optimize n-type doping along with p-type doping [6], [26] by adjusting the ratios between CNT, SDBS, PEI, and other molecules or chemicals. Oxygen barriers could be used to prevent degradation of n-type properties. Eventually, a working combined p- and n-type modules can be fabricated and evaluated for the thermoelectric figure of merit [23]. This work will pave the way for lightweight, non-toxic and flexible thermoelectric cells capable of harvesting thermal energy from the human body, solar cells and a host of other areas where it is currently going to waste as well as cooling energy consuming devices.

## Materials and Methods

Carbon nanotubes, made using chemical vapor deposition by CheapTubes Inc., were used for the experiment. The manufacturer claimed the 90 wt% purity CNTs contain single wall nanotubes whose specifications are the following: outer diameter: 1–2 nm, inner diameter: 0.8–1.6 nm, length: 5–30 µm, ash: <1.5 wt%, multi-wall nanotubes: >5 wt%, amorphous carbon: <3 wt%. The 99 wt% purity CNTs contain approximately 50/50 single- and double-wall tubes whose specifications are the following: outer diameter: 1–2 nm, inner diameter: 0.8–1.6 nm, length: 3–30 µm, ash: 0 wt%, multi-wall nanotubes: <2 wt%.

For each experiment, 60 mg of CNTs were dispersed in 5∼15 ml of deionized water with a prescribed amount of SDBS. Sonication was conducted in two modes. The mixture was sonicated for 30 min using pen-type sonicators (XL-2000 from Misonix and FB 120 from Fisher scientific).

Afterward, a determined amount of aqueous 5-wt% PEI (branched, M.W. 600, 99% from Alfar Aesar) solution was added with a pipette into the dispersion. It has been demonstrated that PEI attaches to nanotubes by physisorption on the tube sidewall [27]. To maximize the occurrence of physisorption and create an even coating of PEI on the nanotubes, the mixture solution was stirred for 48 hours while being maintained at a temperature of 50–60°C. The prescribed amount of PVAc (Vinnapas 401, Wacker chemicals) was subsequently added into the dispersion. The mixture was dispersed by pen-type sonicators for 30 minutes each and then poured into a 5 cm×5 cm×2 cm plastic container for casting. Films of 20–80 µm in thickness were formed as the dispersion dried, in a process that usually took between 24 and 48 hours. Dried samples were then thermally annealed in a vacuum oven at 60°C for 4 hrs in order to remove any water which had permeated the film.

Four series of samples were synthesized. In the first, the weight percent of SDBS was varied while the weight percentages of CNT and PEI were maintained at 20 and 10 wt%, respectively. The second and third series varied PEI wt% while maintaining SDBS at 20 wt% and 40 wt%, respectively, with 20-wt% CNTs. The fourth was for testing air stability with 20 or 40 wt% CNTs in the composites. All weight percentages were determined by measuring the mass of the material on a scale before including it in the sample. The weight percent of Vinnapas was varied in each series to make up whatever difference was left between the total of the other three weight percentages and 100 percent. A summary of the experimental details is included in Table 1.

**Table 1:** List of samples with the concentrations of CNT, SDBS, PEI, PVAc as well as the purity of CNT.

Series	Sample	CNT	SDBS	PEI	PVAc	CNT
	Number	wt%	wt%	wt%	wt%	Purity (%)
1	1	20	20	10	50	90
	2	20	40	10	30	90
	3	20	60	10	10	90
	4	20	20	10	50	99
	5	20	40	10	30	99
	6	20	60	10	10	99
2	1	20	20	5	55	90
	2	20	20	10	50	90
	3	20	20	20	40	90
	4	20	20	40	20	90
	5	20	20	10	50	99
	6	20	20	20	40	99
	7	20	20	30	30	99
	8	20	20	40	20	99
	9	20	20	50	10	99
3	1	20	40	10	30	90
	2	20	40	20	20	90
	3	20	40	30	10	90
	4	20	40	0.1	39.9	99
	5	20	40	1	39	99
	6	20	40	5	35	99
	7	20	40	10	30	99
	8	20	40	20	20	99
	9	20	40	30	10	99
	10	20	40	40	0	99
4	1	20	40	40	0	99
	2	40	40	10	10	90

**The samples were divided into four different series in order to identify the influence of the SDBS concentration (Series 1), the PEI concentration (Series 2 and 3), and air stability (Series 4) on the electrical properties.**

After fabrication, each sample was tested for conductivity and thermopower values. To this end, a rectangular test sample of the dried and annealed film was removed from the plastic container. Conductive silver paint was applied to the sample strip to minimize electrical contact resistance and the relevant dimensions were measured using a micrometer. Details can be found in our previous publications [3], [4], [5], [6].
